# The Big Entity of New RNA World: Long Non-Coding RNAs in Microvascular Complications of Diabetes

**DOI:** 10.3389/fendo.2018.00300

**Published:** 2018-06-04

**Authors:** Satish K. Raut, Madhu Khullar

**Affiliations:** Department of Experimental Medicine and Biotechnology, Postgraduate Institute of Medical Education and Research, Chandigarh, India

**Keywords:** long non-coding RNA, diabetes complications, diabetic retinopathy, diabetic nephropathy, diabetic cardiomyopathy, diabetic neuropathy

## Abstract

A major part of the genome is known to be transcribed into non-protein coding RNAs (ncRNAs), such as microRNA and long non-coding RNA (lncRNA). The importance of ncRNAs is being increasingly recognized in physiological and pathological processes. lncRNAs are a novel class of ncRNAs that do not code for proteins and are important regulators of gene expression. In the past, these molecules were thought to be transcriptional “noise” with low levels of evolutionary conservation. However, recent studies provide strong evidence indicating that lncRNAs are (i) regulated during various cellular processes, (ii) exhibit cell type-specific expression, (iii) localize to specific organelles, and (iv) associated with human diseases. Emerging evidence indicates an aberrant expression of lncRNAs in diabetes and diabetes-related microvascular complications. In the present review, we discuss the current state of knowledge of lncRNAs, their genesis from genome, and the mechanism of action of individual lncRNAs in the pathogenesis of microvascular complications of diabetes and therapeutic approaches.

## Introduction

Diabetes mellitus is a metabolic disorder resulting from a deficiency or failure to maintain normal glucose homeostasis (1). Diabetes is known to be associated with an increased risk of cardiovascular, renal, and cerebrovascular diseases resulting in high morbidity and mortality (2). The deleterious effects of diabetes include both microvascular (diabetes induced nephropathy, cardiomyopathy, neuropathy, and retinopathy) and macrovascular (CAD, peripheral arterial disease, and stroke) complications (1). Diabetes-induced microvascular complications are a consequence of deregulated expression of genes of several molecular pathways involved in their pathogenesis (3).

Long non-coding RNAs (lncRNAs), which are more than ≥200 nucleotides long, are a novel class of functional RNAs that do not code for any proteins. However, several ribosome-associated annotated lncRNAs contain a coding region that translates a peptide (4). lncRNAs regulate expression of several genes by binding to specific DNA/RNA or protein moieties (5). Unlike other small non-protein coding RNAs (ncRNAs), lncRNAs are not well conserved and function through diverse mechanisms (6). They may act (i) as scaffolds to bring protein complexes together, (ii) as sponges for microRNAs, (iii) as host genes for microRNAs, (iv) as controllers of mRNA decay, DNA sequestration of transcription factors, and epigenetic regulation of chromatin compaction, and (v) as stabilizers of mRNA through miRNA binding site masking (6–8).

Long non-coding RNAs have been implicated in a wide range of physiological processes and in the pathophysiology of several diseases, including diabetes and diabetes-induced microvascular complications (9–11). This review discusses the current state of knowledge of lncRNAs, their genesis from the genome, and the mechanism of action of individual lncRNAs in the pathogenesis of microvascular complications of diabetes and their potential as new therapeutics in diabetes-induced microvascular complications.

## lncRNAs and Biogenesis

Nearly 2% of the genome is transcribed into protein coding RNAs and remaining 70–90% is transcribed into ncRNAs (12). Non-coding RNAs such as tRNAs, rRNAs, and spliceosomal RNA are critical components of many cellular machines (13). Apart from these classical ncRNAs, microRNAs and lncRNAs are being recognized as important regulators of gene expression. For a long time, lncRNAs were presumed to be transcriptional “noise” lacking protein coding potentials and with low levels of evolutionary conservation (14). However, in the past decade, strong evidence has emerged indicating that lncRNAs are (i) regulated during various cellular processes, (ii) exhibit cell type-specific expression, (iii) localize to specific organelles, and (iv) associated with human diseases (13). Current advanced transcriptomic technologies such as RNAseq, transcriptomics, and next-generation sequencing have led to the identification of several novel lncRNAs transcripts which seem to be evolved from intronic regions of genes (15). These non-protein coding gene loci transcribe upto 50,000 lncRNAs in humans and are poorly conserved in other species. There is uncertainty about how many of these lncRNAs are functional (16). Based on their genomic location, lncRNAs have been classified into five subclasses: intergenic lncRNAs, transcription start site-associated RNAs, intronic lncRNAs, natural antisense transcripts (NATs), and some transcribed pseudogenes (17) (Table 1).

**Table 1:** Classification of long non-coding RNAs (lncRNAs) based on genomic location.

lncRNA	Genomic context	Examples
Intergenic lncRNAs	Transcribed inter genetically from both strands by RNA Pol II	*Xist, H19, HOTAIR, MALAT1*

Intronic lncRNAs	Originates from introns of protein coding genes	*COLDAIR, PPP3CB, MAP3K14* and *DAPK1 loci*

Transcription start site-associated lncRNAs	Transcribed from promoter upstream region and enhancer region by RNA pol II	Associated with *EXT1* and *RBM39* genes, associated with *RNAF12* and *CCDC52* genes

Natural antisense transcripts	Transcribed from the opponent strand of either protein coding or non-protein coding genes	*Xist/Tsix, Kcnq1/Kcnq1ot1, Igf2r/Air*

Pseudogene lncRNAs	Residues of their parental genes that lost encoding function due to mutations	*Xist* evolved from protein-coding gene *Lnx3*

## Intergenic lncRNAs

These are large intervening distinct ncRNAs or lincRNAs, which are found in sequence spaces and do not overlap protein-coding genes. Most of the intergenic lncRNAs, characterized till now, are found to be transcribed by RNA polymerase II and give rise to splice, poly A containing lncRNAs and are of average length of approximately 1,000 bp. Examples are *Xist, H19, HOTAIR*, and *MALAT1* (15).

## Transcription Start Site-Associated lncRNAs

The short-lived medium-length lncRNAs (200–2,000 nt) are transcribed from promoter upstream region and enhancer region by RNA pol II (18). These short transcripts (promoter-associated transcripts), i.e., PROMPTs are processed from the promoter upstream transcripts (15). These transcripts have protein coding like features such as 5′ capping and poly A tailing and occurs in low copy number and easily get degraded by exosomes (15). The function of promoter-associated transcripts is not known. It is not clear whether they have some regulatory function or these are simply a transcriptional by-product (18). The short transcripts (eRNAs) of average length less than 2 kb are the bidirectional transcripts. However, their processivity and biological function are also not known (15).

## Intronic lncRNAs

These consist of small ncRNAs, known to originate from introns of protein-coding genes. Recent studies show that lncRNAs are also encoded within the introns of annotated genes (19). For example, lncRNAs *COLDAIR* has been shown to be involved in plant vernalization and located in the first intron of *FLC*; a flowering repressor locus (15, 20). These lncRNAs have been shown to have a role in various cellular and pathological processes. For example, many of these lncRNAs have been found to be differentially expressed in cancer (15).

## Natural Antisense Transcripts

These lncRNAs get transcribed from the opponent strand of either protein coding or non-protein coding genes, in the genome and modulates the function of sense strand of the gene, and/or several other genes (21). These, too, have features like protein coding RNAs, such as splicing, 5′ capping, and polyadenylation and were originally discovered in bacteria (15). The well known examples of NATs are (i) *Xist*/*Tsix* which control X chromosome inactivation (22) and (ii) *Kcnq1/Kcnq1ot1* and *Igf2r/Air* which are involved in imprinting (15, 23, 24).

## Pseudogene lncRNAs

Pseudogenes are residues of their parental genes that have lost their encoding function due to nonsense, frameshift, and other mutations (25). Some pseudogenes are transcribed into lncRNAs of more than 200 nt in length and have high levels of sequence conservation (26). Two types of pseudogenes are known: (a) expressed pseudogenes which are intermediates on their way to complete pseudonization and (b) dead pseudogenes that have adopted new mutations (15). An example of pseudogene is the *Xist* lncRNAs which have evolved from the protein-coding gene *Lnx3*, by pseudogenization (27).

## Diabetes-Related lncRNA

Diabetes mellitus includes (i) type 1 diabetes mellitus (T1DM), (ii) type 2 diabetes mellitus (T2DM), (iii) gestational diabetes mellitus, and (iv) other specific types of diabetes mellitus (28). Type I diabetes is also known as insulin-dependent diabetes and occurs commonly in childhood and early adulthood. Although the exact etiology of T1DM is not known, it is considered to be primarily due to genetic factors and involves immuno-destruction of insulin producing pancreatic B cells. T2DM is adult onset, non-insulin-dependent DM and is the most common type of diabetes, exceeding 90% of all cases of diabetes and is attributed to environmental and genetic factors (28). lncRNAs have recently gained widespread attention in a variety of human diseases including diabetes. Aberrant expression of several lncRNAs has been observed in both T1DM and T2DM. Some of the altered lncRNAs were found to be common in both. Table 2 shows the list of deregulated lncRNAs and their functions in different types of DM.

**Table 2:** Long non-coding RNA (lncRNA) associated with various types of diabetes.

lncRNAs	Findings	Diabetes type	Reference
ERBB3 locus-associated lncRNA (NONHSAG011351)	Prevent β-cell destruction	Type 1 diabetes	(69)

Plasmacytoma variant translocation 1	Associated with end stage renal disease attributed to type 1 diabetes mellitus (T1DM)	Type 1 diabetes	(70)

Maternally expressed 3 gene	Alters susceptibility to T1DM	Type 1 diabetes	(71, 72)
	Regulates β cell identity and function *via* insulin production and apoptosis in mouse MIN6 cells and isolated mouse islets		

	Associated with impaired glucose tolerance, and insulin synthesis and secretion	Type 2 diabetes	(73)

HI-LNC25	Positively regulates *GLIS3* (which contains both T1D and T2D risk variants) in EndoC-βH1 human β cell line	Type 1 diabetes	(74)

β-cell long intergenic non-coding RNA (βlinc1)	Regulates β cell identity and function in mouse MIN6 cells and EndoC-βH1 human β cell line; also regulates its neighboring gene NKX2.2 (an islet transcription factor)	Type 1 diabetes	(75)

	Associated with β-cell loss	Type 2 diabetes	(75)

TUNAR (HI-LNC78)	Knockdown of *TUNAR* leads to impaired glucose-stimulated insulin secretion in human islets	Type 1 diabetes	(76)

PLUT (HI-LNC71)	Regulates transcription of *PDX1*, a key pancreatic β cell transcriptional regulator, in EndoC-βH1 cells, primary islet cells, mouse β cell line MIN6	Type 1 diabetes	(76)

MALAT1	Upregulation of *MALAT1* is associated with microvascular dysfunction (diabetic retinopathy) in STZ-induced diabetic rats and db/db mice	Type 1 diabetes	(53)

TUG1	Downregulation of lncRNA TUG1 expression increased apoptosis and reduced insulin secretion in mouse β cells	Type 1 diabetes	(77)

NONRATT021972	Exacerbated neuropathic pain *via* TNF-α related pathways	Type 2 diabetes	(78)

ENST00000550337.1	Biomarker	Pre-diabetes and Type 2 diabetes	(79)

lncRNA-ROR	Maintenance of human amniotic epithelial stem cell pluripotency and β islet-like cell differentiation	Type 2 diabetes	(80)

Antisense non-coding RNA (ANRIL)	Affect β-cell mass	Type 2 diabetes	(81)

E330013P06 (E33)	Promotes macrophage inflammation	Type 2 diabetes	(82)

Imprinted maternally expressed transcript (H19)	Associated with increased birth weight; higher expression in T2D patients	Type 2 diabetes	(83)

HI-LNC901	Implicated in islet function	Type 2 diabetes	(84)

Nuclear paraspeckle assembly transcript 1	Regulates mTOR signaling pathway	Type 2 diabetes	(85)

PDX1-associated lncRNA, upregulator of transcription (PLUTO)	Regulates PDX1 expression	Type 2 diabetes	(76)

## lncRNA and Microvascular Complications of Diabetes

Recent studies have shown dysregulation of several lncRNAs in diabetic nephropathy (DN), retinopathy, neuropathy, and cardiomyopathy suggesting their potential role in the pathophysiology and hence a potential therapeutic target of diabetes-induced microvascular complications (19).

## lncRNAs and Diabetic Nephropathy

**Figure 1:**
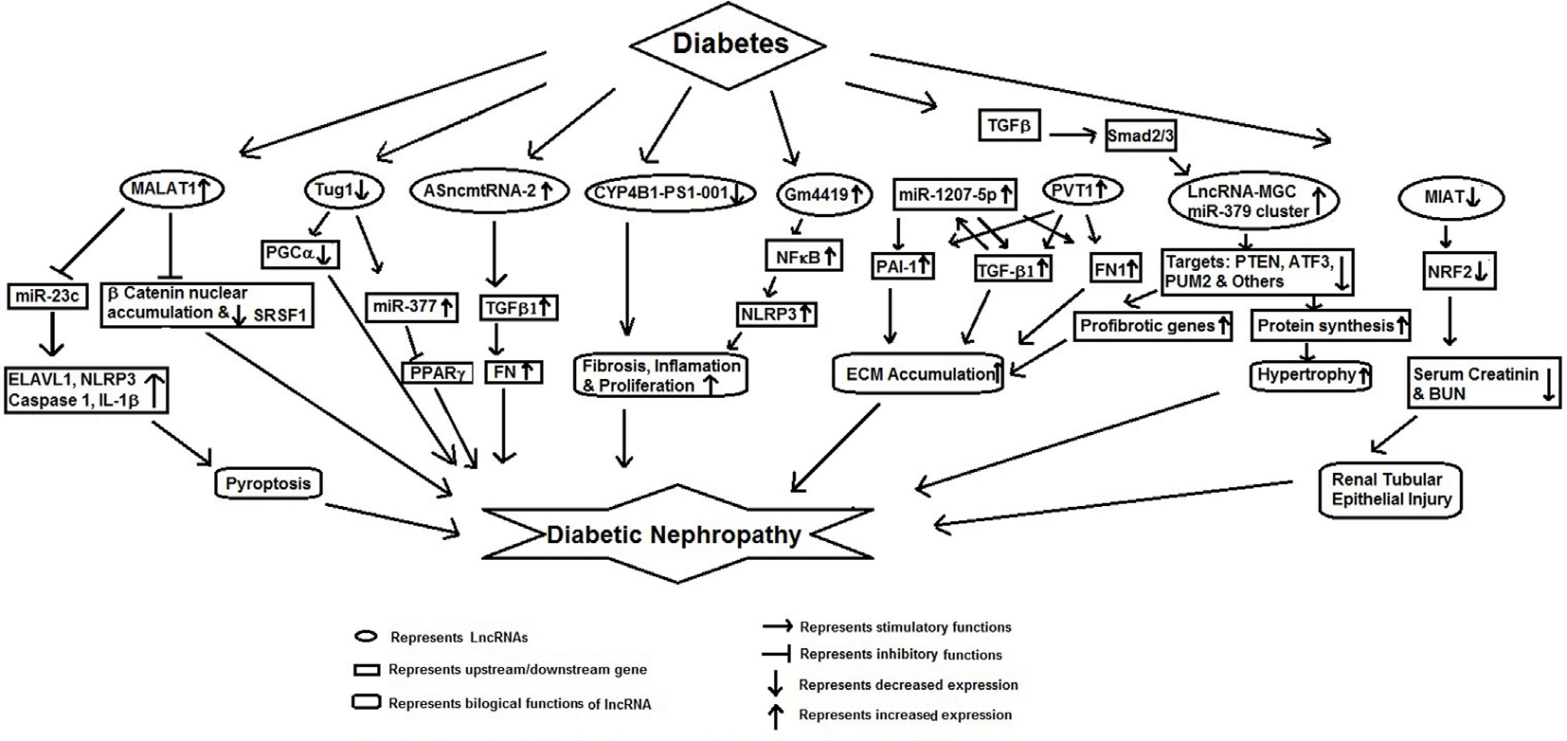
Schematic model showing functional role of long non-coding RNAs in diabetic nephropathy.

Several lncRNAs have been shown to regulate epigenetic changes and associated metabolic memory in DN (Figure 1). It has been suggested that DN may induce differential expression of lncRNAs in renal tissues leading to deregulation of multiple molecular pathways involved in its pathophysiology. For example, Chen et al. showed differential expression of 311 altered lncRNAs in a mouse model of DN as compared to the db/m control mouse (29). These lncRNAs were found to target several different pathways enriched in glutathione metabolism signaling, indicating lncRNAs may deregulate expression of several DN related pathways. In another study, Wang et al. have also found differential expression of 1,018 lncRNAs in the kidney tissues of db/db mice having DN. Among these differentially expressed lncRNAs, *CYP4B1-PS1-001* was significantly downregulated. The overexpression of *CYP4B1-PS1-001* suppressed proliferation and fibrosis of mesangial cells (MCs). All these findings suggest the pivotal role of *CYP4B1-PS1-001* in the proliferation and fibrosis of mice MCs (30).

Long non-coding RNA metastasis-associated lung adenocarcinoma transcript 1 (*MALAT1*) is expressed in a variety of cells. An increased expression of *MALAT1* has been reported in diabetic rat (31) and STZ-induced mouse model of DN (32). Li et al. observed an increased renal *MALAT1* expression in STZ-induced diabetic rats and in hyperglycemia (HG) exposed HK-2 cells. The increased *MALAT1* expression was associated with an increased pyroptosis of the renal epithelial cells and decreased expression of miR-23c. Further downregulation of *MALAT1* resulted in the decreased expression of *ELAVL1, NLRP3, Caspase-1* and the pro-inflammatory cytokine IL-1β, and upregulated miR-23c expression and inhibited pyroptosis. These results suggested that upregulation of *MALAT1* was promoting renal cell apoptosis and DN by targeting miR-23c and its downstream target *EVAL1*. The role of *MALAT1* in DN was further confirmed by Hu et al. who showed increased renal cortical *MALAT1* levels in diabetic mice. Mouse podocytes showed an initial increase in *MALAT1* followed by a decline. The decrease in *MALAT1* was followed by translocation of β-catenin to the nucleus and increased expression of *MALAT1* RNA-binding protein, serine/arginine splicing factor 1 (SRSF1) (32). Inhibition of *MALAT1* corrected the podocyte damage *via* suppressing an *MALAT1* lncRNA-binding protein (SRSF1). *MALAT1* levels were found to be under the regulation of β-catenin and knockdown of β-catenin led to decreased *MALAT1* levels (32). These results provide evidence for a potential role of *MALAT1* in DN.

Antisense mitochondrial non-coding RNA-2 (*ASncmtRNA-2*) has been also shown to be upregulated in the animal model of DN and in high glucose treated MCs human renal mesangial cells. Gao et al. showed that the expression of *ASncmtRNA-2* could be suppressed by nitric oxide synthase inhibitor, NG-nitro-l-arginine methylester (L-NAME). Furthermore, *ASncmtRNA-2* upregulation was accompanied by increase in *TGFβ1* and its downstream gene, *fibronectin*. These authors have proposed that ROS mediated upregulation of *ASncmtRNA-2* promotes DN through increased transcription of pro-fibrotic factors (33).

Yi et al. reported differential expression of 14 lncRNAs in renal tissues from db/db diabetic mice with nephropathy and in MCs in response to HG (34). Among these, LincRNA-*Gm4419* showed *NF-κB* as its target, suggesting its role in renal fibrosis. Knockdown of *Gm4419* inhibited the expression of pro-inflammatory cytokines, biomarkers of renal fibrosis, and reduced cell proliferation in MCs, suggesting that lincRNA-*Gm4419* promoted MC inflammation, fibrosis and proliferation hyperglycemic condition through NF-κB/NLRP3 inflammasome pathway (34).

Long non-coding RNA taurine-upregulated gene 1 (*Tug1*) regulates PPARγ coactivator α (PGC-1α, encoded by Ppargc1a) and was shown to be differentially expressed in glomeruli from diabetic milieu (35). Overexpression of this lncRNA was associated with marked improvement in biochemical and histological features of diabetic mice along with elevated expression of PGC-1α and its downstream target genes, suggesting that *Tug1* acts by targeting mitochondrial bioenergetics in the podocyte cells of diabetic mice (35). Duan et al. have also recently shown that *Tug1* ameliorated ECM accumulation through microRNA-377, targeting *PPAR*γ in DN (36).

One of the major characteristic of DN is the excessive accumulation of ECM in the kidney glomeruli. Alvarez and DiStefano reported that lncRNA plasmacytoma variant translocation 1 (*PVT1*) was mediating ECM accumulation in glomeruli in DN (37). These authors showed that *PVT1* regulated the expression of ECM-related proteins (TGF-β1, PAI-1, and FN1). Their findings suggest that *PVT1* contributes to the progression of DN by regulating ECM expression (37). Alvarez et al. reported that miR-1207-5p is derived from *PVT1* lncRNA and was upregulated by high glucose and TGFβ1 in kidney cells. Further they showed that like *PVT1*, miR-1207-5p also increased the expression of *TGF-β1, PAI-1*, and *FN1* independently of *PVT1*. This indicates that glucose and TGFβ1 regulates miR-1207-5p expression but in an independent manner of its host gene *PVT1*. Taken together, these results show that miR-1207-5p and its host gene play an important role in the pathogenesis of DN (38).

Genotype loci rs13447075 and rs2648862 have been reported to strongly associated with diabetes-induced end stage renal disease (ESRD) (39). rs13447075 is situated in the coding portion of one of the transcript variants of *PVT1*, indicating an association of *PVT1* in mediating susceptibility to ESRD attributable to diabetes (39).

Wang et al. observed decreased renal cortical expression of lncRNA *ENSMUST00000147869* in db/db and db/m mice which was associated with Cyp4a12a levels (40). Overexpression of this lncRNA in mouse MCs rectified the fibroblast proliferation and fibrosis indices, suggesting a role of *ENSMUST00000147869* in MC proliferation and fibrosis and as a potential biomarker of DN (40). Kato et al. have reported an increased expression of 40 microRNAs and their host lncRNA transcript (*lnc-MGC*) in glomeruli and in MC’s exposed to TGFβ1 or high glucose. Furthermore, a decreased expression of cluster microRNAs and *lnc-MGC* was seen in diabetic Chop−/− mice, which conferred protection from DN. Knockdown of *lnc-MGC* inhibited the expression of cluster microRNAs, and also decreases ECM and hypertrophy in diabetic mice (41). They proposed that *lnc-MGC* could be used as a therapeutic target for controlling the progression of DN. Zhou et al. have examined the role of lncRNA-myocardial infarction-associated transcript (*MIAT*) in diabetes-induced renal tubular injury. They found decreased expression of both *MIAT* and *Nrf2* in diabetes-induced renal tubule, as well as in HG exposed HK-2 cells, which inversely correlated with serum creatinine and BUN. Overexpression of *MIAT* reversed inhibitory action of high glucose induced *Nrf2* expression, indicates that *MIAT/Nrf2* axis acts as an important signaling pathway for HG induced renal tubular epithelial injury (42).

## lncRNAs and Diabetic Retinopathy (DR)

**Figure 2:**
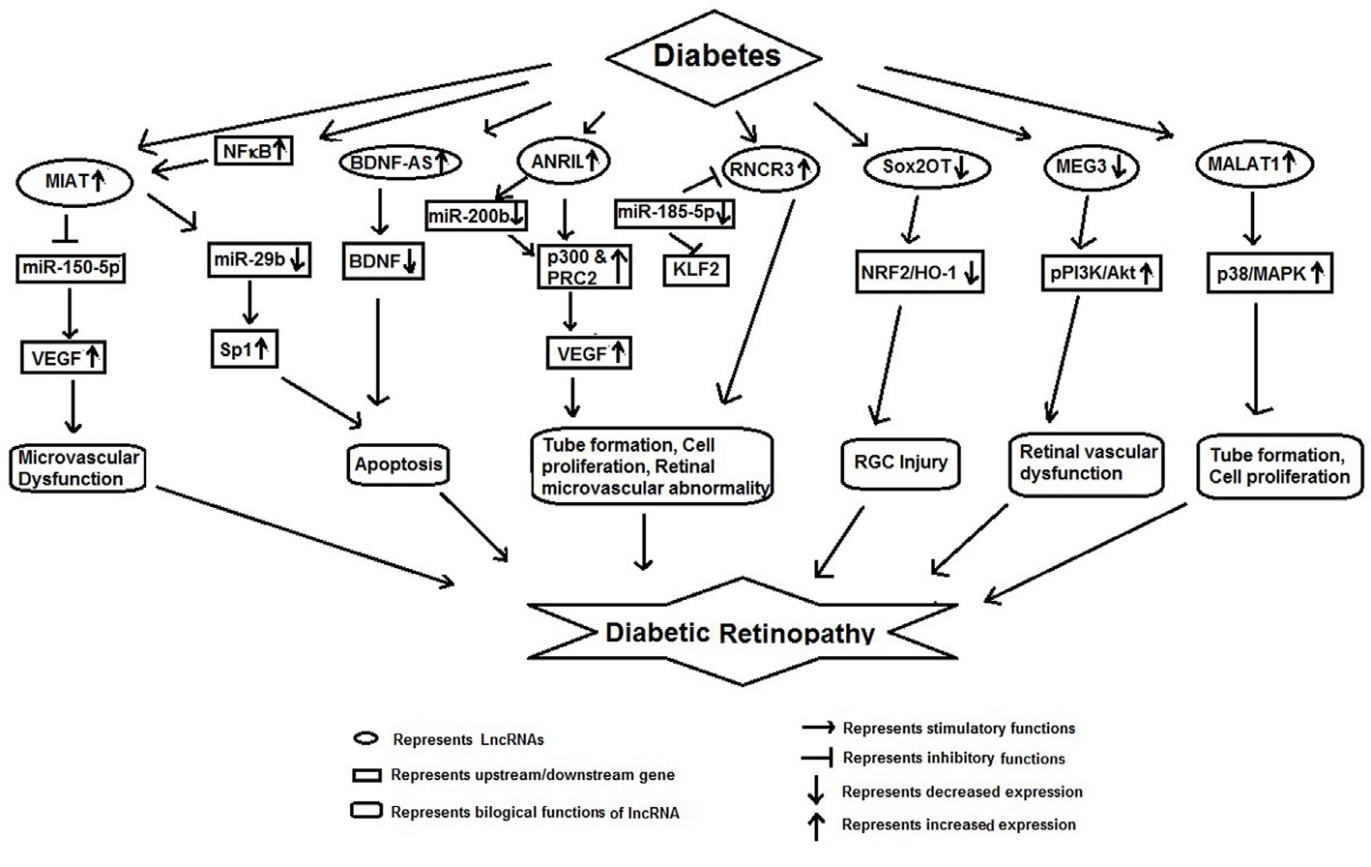
Schematic model showing functional role of long non-coding RNAs in diabetic retinopathy.

Diabetic retinopathy is a major complication of diabetes and leads to vision loss globally (43). Recent studies show that lncRNAs may be involved in its pathophysiology thus adding another dimension to its therapeutic targeting (Figure 2).

Yan et al. demonstrated that high glucose augmented *MIAT* expression in diabetic retinas and endothelial cells. Silencing *MIAT* improved diabetes-induced retinal microvascular dysfunction *in vivo*. Similarly, it inhibited endothelial cell proliferation, migration, and tube formation *in vitro*. Further *MIAT* was found to be a competing endogenous RNA, which formed a feedback loop with VEGF and miR-150-5p to regulate endothelial cell function (44).

Apoptosis is a prominent feature of DR. Recently Zhang et al. showed that *MIAT* mediates the cellular apoptosis regulatory network. These authors observed an increased expression of *MIAT* and *NF-*κ*B* (*p-p65*) in DR. They observed that NF-κB directly binds with *MIAT*, and treatment of rCM-1 cells with Bay11-7082 inhibited binding between NF-κB and *MIAT*, suggesting that Bay11-7082 acted as an inhibitor for NF-κB which suppressed the *MIAT* binding activity. Moreover, *MIAT* was shown to be regulated by miR-29b. Furthermore, *MIAT* inhibition significantly reversed the high glucose induced low expression of miR-29b, high expression of *Sp1* (target of miR-29b) and apoptosis. These findings suggest that *MIAT* regulates cell apoptosis *via* its association with NF-κB (p-p65), and *miR-29b* (45).

Li et al. have recently reported that lncRNAs, brain-derived neurotrophic factor antisense (*BDNF-AS*) was upregulated whereas *BDNF* was markedly downregulated in human RPE cell lines in response to HG. Inhibition of *BDNF-AS* ameliorated d-glucose induced apoptosis and upregulated *BDNF* in ARPE-19 cells, suggesting that *BDNF-AS*, plays a critical role in the process of glucose induced apoptosis in DR through inverse regulation of *BDNF* (46).

Hyperglycemia increases the expression of lncRNA *ANRIL* in the retina and in retinal endothelial cells (47). This increased expression of *ANRIL* is responsible for altered expression and function of VEGF. In glucose-exposed ECs and in the retinal tissue of diabetic animals, *ANRIL* regulated glucose-mediated upregulation of VEGF through its interaction with p300 and PRC2 components. Inhibition of *ANRIL* in HRECs and in ANRILKO mice, significantly reduced VEGF *EZH2* and *p300* mRNA expression (47). These results suggest potentially new targeted method to prevent DR using an RNA-based approach in DR.

Like apoptosis, retinal microvascular abnormality is also an important pathological feature of DR (48). Shan et al. have shown HG-induced *RNCR3* upregulation *in vivo* and *in vitro*. They further demonstrated that endothelial cell function was regulated through RNCR3/KLF2/miR-185-5p regulatory network. Knockdown of *RNCR3* decreased cell proliferation, migration, and tube formation *in vitro* and was shown to alleviate vascular dysfunction in retina. Similarly, Liu et al. have also shown that knockdown of *RNCR3* significantly inhibited retinal reactive gliosis. Collectively, these observations suggest that *RNCR3* knockdown may be a potential therapeutic molecule for the prevention of diabetes-induced retinal microvascular abnormalities (48, 49).

Retinal ganglion cell (RGC) injury is also one of the distinguished pathological features of DR (50). Li et al. reported decreased expression lncRNA *Sox2OT* in the retinas of STZ-induced diabetic mice as well as RGCs in the presence of high glucose or oxidative stress. Inhibition of *Sox2OT* was found to protect high glucose-induced RGCs *in vitro* and appeared to play a neuroprotective role in diabetes-related retinal neurodegeneration *in vivo*. *Sox2OT* knockdown also regulated NRF2/HO-1 signaling activity, indicating that it also had anti-oxidant function. Thus, *Sox2OT* knockdown may be a potential treatment option for diabetes-induced retinal neurodegeneration (50).

Similarly, high glucose and oxidative stress were found to downregulate lncRNA-*MEG3* in the retinas of STZ-induced diabetic mice and endothelial cells. Inhibition of *MEG3* aggravated retinal vessel dysfunction *in vivo*, and also regulated retinal endothelial cell proliferation, migration, and tube formation *in vitro* (51). These studies suggest that overexpression of *MEG3* may act as a therapeutic target for the treatment of DR.

Yan et al. have reported aberrant ocular expression of nearly 300 lncRNAs in diabetic mice. Among these 214 lncRNAs were downregulated and 89 lncRNAs upregulated. lncRNA *MALAT1* was significantly increased in various models of diabetic mellitus, such as high glucose treated RF/6A cell, in the samples of aqueous humor, and in fibrovascular membranes of diabetic patients (52). lncRNA *MALAT1* has been shown to play a pathogenic role in diabetes-induced endothelial cell dysfunction and its inhibition was found to inhibit retinal endothelial cell proliferation, migration, and tube formation and ameliorate DR (53). These authors suggested that crosstalk between the *MALAT1* and p38 MAPK signaling pathway was involved in regulating retinal endothelial cell function and silencing *MALAT1* had potential in anti-angiogenic therapy in DR (53).

## lncRNAs and Diabetic Neuropathy (DNP)

**Figure 3:**
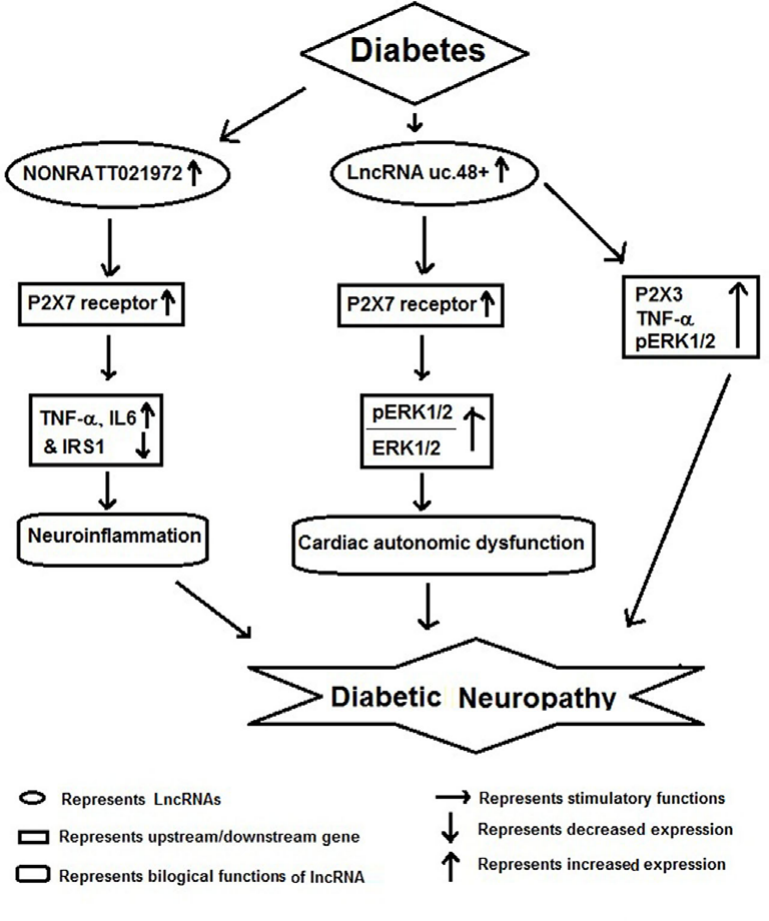
Schematic model showing functional role of long non-coding RNAs (lncRNAs) in diabetic neuropathy.

Diabetic neuropathy is also a chronic complication of diabetes and has potential life-threatening consequences (54). Several reports have shown that lncRNAs act as important players in the pathophysiology of DNP (Figure 3). For example, Xu et al. reported increased expression of *NONRATT021972* lncRNA in sympathetic neuronal-like PC12 cells in response to HG and high free fatty acid (HF) exposure. Inhibition of this lncRNA in PC12 cells significantly relieved HG-HF-induced tumor necrosis factor-α, interlukin-6, and recovered cell viability mediated by P2X7 receptor. They also observed increased expression of *NONRATT021972* lncRNA in superior cervical ganglia of diabetic rats and found that treatment with *NONRATT021972* siRNA led to decrease the expression of *TNF-α*, blocked serine phosphorylation of insulin receptor substrate (IRS) 1 and increased *IRS1* expression in SCG of diabetic rats (55). These results suggest that inhibition of *NONRATT021972* lncRNA could be targeted for prevention of DNP (54, 55).

Recent studies show that DNP results in differential expression of several lncRNAs in SCG, which may result in deregulation of multiple molecular pathways involved in its pathophysiology. Li et al. found a differential expression of several lncRNAs regulating gene expression of pathways involved in immune response, cell migration, defense response, taxis, and chemotaxis. According to Kyoto Encyclopedia of Genes and Genomes, many of the target genes of differentially expressed lncRNAs were located in cytokine–cytokine receptor interactions, chemokine signaling pathway, and cell adhesion molecules. Furthermore, by gene co-expression network analysis, these authors showed 268 regulation edges among 105 lncRNAs and 11 mRNAs. These observations show that there is a co-expression of specific lncRNAs along with their target mRNAs in diabetic cardiac autonomic ganglia. In conclusion, authors have suggested a possible role for multiple lncRNAs as potential therapeutic targets or biomarkers for diabetic cardiac autonomic neuropathy (56).

A similar study by Wu et al. also found that several lncRNAs were differentially expressed in SCG of diabetic rats and among them uc.48+ lncRNA was significantly increased. The increased expression of uc.48+ lncRNA and P2X7 receptor was associated with the cardiac dysfunction. Silencing *uc.48*+ alleviated cardiac dysfunction, decreased its target gene P2X7 and the ratio of phosphorylated extracellular regulated protein kinases1/2 to extracellular regulated protein kinases1/2 in SCG of diabetic rats. Furthermore, they observed that silencing *uc.48*+ lncRNA improved diabetic sympathetic neuropathy through P2X7 and ERK signaling pathway (57). lncRNA *uc.48*+ inhibition has been also shown to decrease TNF-α and *P2X3* expression and activation of ERK1/2 in the DRG of DM rats, indicating that *uc.48*+ inhibition can alleviate the DNP by inhibiting the excitatory transmission mediated by the P2X3 receptor in DRG (58).

## lncRNAs and Diabetic Cardiomyopathy

**Figure 4:**
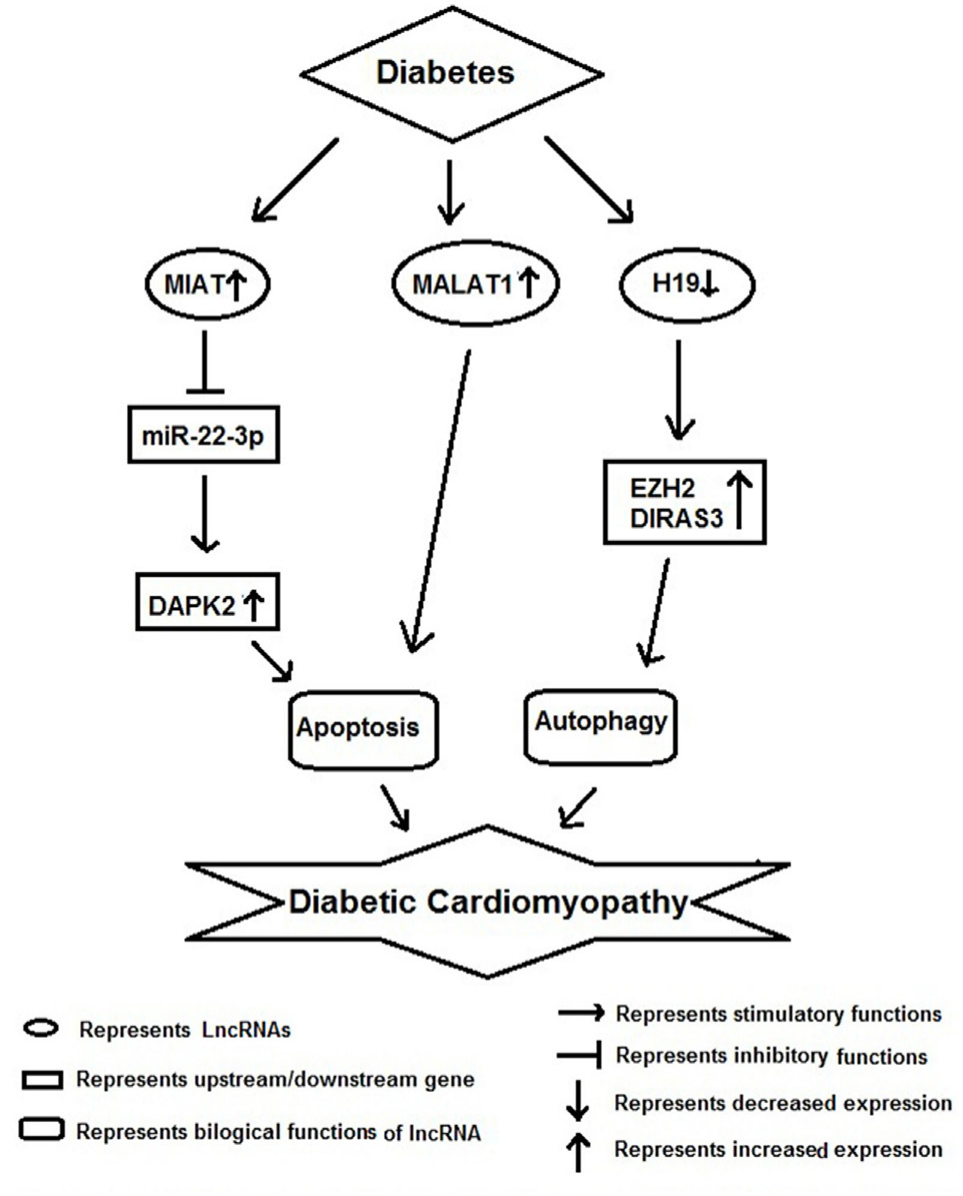
Schematic model showing functional role of long non-coding RNAs (lncRNAs) in diabetic cardiomyopathy.

Diabetic cardiomyopathy along with vascular inflammation is a characteristic feature of diabetes and the key cells involved in these pathological conditions are monocyte/macrophages and vascular smooth muscle cells (Figure 4). Recently, Kesherwani et al. have reported that several coding and non-coding RNAs (including lncRNA) were differentially expressed in insulin 2 mutant (Ins2±) Akita mouse model of diabetes (59). They performed global gene expression profiling and found that 488 transcripts were differentially expressed, including 12 non-coding RNAs. Among these, bone morphogenic protein-10 (*BMP10*) and vomeronasal-1 receptor-180 (*VMN1R180*) were highly upregulated genes; whereas hairy and enhancer of split-related (*HELT*) and WD repeat domain 83 opposite strand (*WDR83OS*) were the most downregulated genes. In addition, they have noticed that *H19* lncRNA and miR-101c were found to be significantly upregulated; whereas *Neat1* lncRNA was the most downregulated in Akita heart (59). In conclusion, they revealed that these results could be used as a platform to initiate focused future studies for the identification of novel therapeutic molecule in DbCM. HG has been shown to induce *MIAT* expression and apoptosis *in vitro* and *in vivo* model of diabetic cardiomyopathy. Furthermore, *MIAT* and *DAPK2* were shown to be a target of miR-22-3p by luciferase assay. Overexpression of *MIAT* antagonized the inhibitory effect of miR-22-3p on *DAPK2*. Knockdown of *MIAT* led to the decrease of *DAPK2* expression, apoptosis, and improves cardiac function in diabetic rats. Their finding suggests that *MIAT* functions as a competing endogenous RNA to upregulate *DAPK2* expression by sponging miR-22-3p in DbCM (60).

In another study, Zhang et al. found an increased expression of *MALAT1* in the heart tissue of diabetic rats. Knockdown of *MALAT1* showed improvement in left ventricular function, partly through the attenuation of cardiomyocyte apoptosis (61). Zhuo et al. have shown decreased expression of *H19* in hearts of diabetic rats, as well as in neonatal cardiomyocytes exposed to high glucose. As a result of it there was an increase in autophagy was noticed *in vitro*. Their RNA-binding protein immunoprecipitation studies showed direct binding of *H19* with EZH2 in cardiomyocyte cells. Augmentation of *H19* in cardiomyocytes in the presence of high glucose resulted in decrease *DIRAS3* expression, suppress autophagy activation, and promote mTOR phosphorylation. In conclusion, they suggested that lncRNA *H19* suppresses autophagy activation by epigenetically silencing *DIRAS3* in DbCM (62).

Long non-coding RNAs have been suggested as circulating biomarker in DbCM (63). These authors reported that circulating lncRNAs such as long intergenic predicting cardiac remodeling (*LIPCAR*), *MIAT*, and smooth muscle and endothelial cell-enriched migration/differentiation-associated long non-coding RNA (*SENCR*) were found to be affiliated with grade I diastolic dysfunction. Further these lncRNAs were validated in a sample of 30 patients of diabetes and normal subjects. Indicating that these lncRNAs are independent biomarkers for diastolic function and remodeling in diabetic patients.

Angiotensin II is an important vasoconstrictor and pro-fibrotic and proliferative peptide. In vascular smooth muscle cells, several lncRNAs have shown to be deregulated by angiotensin II signaling pathway (64). For example, angiotensin II was shown to increase *lnc-Ang362* expression in vascular smooth muscle cells. This induction of *lnc-Ang362* increased expression of two miRNAs (mir-221 and mir-222) and cell proliferation. This suggests that *lnc-Ang362* has an important role in vascular smooth muscle cell proliferation (65). In human vascular smooth muscle cells, lncRNA *SENCR* was involved in regulation of both contractile genes and genes involved in regulation of MYOCD (66, 67). Recently, Ballantyne et al. reported lncRNA *SMILR* in atherosclerotic plaques and its inhibition decreased vascular proliferation, suggesting this lncRNA promoted cellular proliferation (68).

## Conclusion

Long non-coding RNA are important regulators of gene expression and control gene expression by binding to specific cellular moieties. The discovery of lncRNAs adds a new layer of complexity to the molecular etiology of microvascular complications of diabetes. Several lncRNAs have been identified and implicated in microvascular complications of diabetes by deregulating pathways, such as fibrosis, oxidative stress, endothelial dysfunction, etc. However, the mechanisms of many of deregulated lncRNAs are yet to be delineated. Thus, further research focusing on their mode of action in disease etiology and pathology is needed to understand their role in the pathogenesis of diabetes. A better understanding the mechanisms of newly identified lncRNAs can pave the way for early diagnosis and the design of better therapeutics.

## Author Contributions

SR: data collection, writing, figure preparation, and references. MK: data collection, writing, editing, figure preparation, and references.

## Conflict of Interest Statement

The authors declare that the research was conducted in the absence of any commercial or financial relationships that could be construed as a potential conflict of interest.
